# Temporal Changes in Esophageal Cancer Mortality by Geographic Region: A Population-based Analysis

**DOI:** 10.7759/cureus.3596

**Published:** 2018-11-15

**Authors:** Bhargava Chitti, Anthony Pham, Stephen Marcott, Xin Wang, Louis Potters, A. Gabriella Wernicke, Bhupesh Parashar

**Affiliations:** 1 Radiation Oncology, New York-Presbyterian Weill Cornell University Hospital of Columbia and Cornell, New York, USA; 2 Radiation Oncology, Los Angeles County General / Keck School of Medicine of the University of Southern California, Los Angeles, USA; 3 Radiation Oncology, Weill Cornell Medical Center, New York, USA; 4 Radiation Oncology, NewYork-Presbyterian/Weill Cornell Medical Center, New York, USA; 5 Radiation Oncology, Zucker School of Medicine at Hofstra / Northwell, New York, USA; 6 Radiation Medicine, Zucker School of Medicine at Hofstra / Northwell, New York, USA

**Keywords:** esophageal cancer, seer, geographic analysis, adenocarcinoma, squamous cell carcinoma

## Abstract

Purpose

To analyze differences in esophageal cancer survival by geographic region of the U.S. from the 1970s to the 2000s, and attribute the causes of these discrepancies.

Methods

Raw data were obtained from the Surveillance Epidemiology and End Results (SEER) program of the National Cancer Institute. Patients were stratified by decade of diagnosis and by geographic region (East, Hawaii/Alaska, Midwest, Southwest, and West), containing SEER registries. The Kaplan-Meier method with the log-rank test was used to compare the overall survival (OS) among these geographic groups. A multivariate Cox Proportional Hazard analysis was conducted to evaluate the impact of the following factors on differences in survival: patient age, gender, race, tumor stage, site, histology, treatment method, and metropolitan size.

Results

A total of 87,834 patients were identified. OS has increased significantly since 1973, with five-year OS improving from 4.9% (the 1970s) to 15.3% (2000s) (*P*<0.001). Residence in the East was prognostic for higher OS compared to all the other regions, with a median OS of six months in the 1970s and 12 months in the 2000s (*P*<0.001). The multivariate analysis revealed increased age, African American race, distant disease, non-distal tumor location, squamous cell histology, and no radiation therapy were associated with worse OS. The West and East had the highest amount of cancer centers (12 and seven, respectively). And the East had the highest number of cancer centers per person (5.7E-07) while the South had the lowest (1.6E-07).

Conclusions

There are disparities in esophageal cancer survival and quality of care through different geographic regions of the U.S., which may be attributed to a combination of the unbalanced distribution of medical resources, the regional differences in cancer biology, and other lifestyle and socioeconomic factors. More research should be conducted to further characterize regional differences and guide the implementation of improvements in survival.

## Introduction

Since the 1970s, overall survival (OS) for esophageal cancer in the U.S. has increased, due in part to advances in treatment modalities and methods [1-2]. Still, in contrast to the general cancer patient population, the prognosis for patients with esophageal cancer remains poor, as indicated by the increase in mortality and incidence since 1975 [3-5]. It is estimated that in 2018, there will be 17,290 new cases of esophageal cancer, and 15,850 deaths due to the disease, within the U.S. [6]. Many factors may be responsible for different survival rates across populations. Notably, risk factors for esophageal cancer vary by histology with the two main types being squamous cell carcinoma (SCC) and esophageal adenocarcinoma. Historically, squamous cell carcinoma (SCC) was the most common histology of esophageal cancer, but recently in western nations, esophageal adenocarcinoma rates of incidence and mortality are rising [7-9]. The known risk factors that predispose to SCC include tobacco smoking, excessive alcohol consumption, caustic injury, nutritional deficiencies, and poor oral hygiene. Risk factors for adenocarcinoma include increased age, male gender, tobacco smoking, gastroesophageal reflux disease (GERD), and dietary deficiencies in fruit and vegetables. Globally, residence in an underdeveloped nation increases the risk for esophageal cancer, with 81% of new diagnoses of esophageal cancer and 82% of mortality in 2012 occurring in underdeveloped nations. In males, esophageal cancer occurred most frequently in Eastern Asia, Southern Africa, and Northern Europe, and least frequently in Western and Central Africa, as well as Western Asia. Rates of esophageal cancer were lower in females overall versus males (7.7 vs 3.7 age-specific mortality per 100,000 person-years). In females, esophageal cancer rates were highest in middle Africa, Western Africa, Eastern Asia, and Eastern Africa, and lowest in Micronesia/Polynesia, Southern Europe, Central America, and South-Eastern Asia. Previous studies have already examined the influence of gender, age, race, disease stage, and histology [7-8,10]. Others have examined the influence of gender and age on esophageal cancer and attributed the high male to female esophageal adenocarcinoma ratio before the age range of 60-64, followed by a decrease in the ratio, as a consequence of the pre-menopausal protective effect of estrogen [11].

Residence in specific geographic regions may contribute to differing morbidity and mortality for esophageal cancer patients. Lifestyle, standards of care, and other factors that play a role in the onset and advancement of the disease are not uniform throughout the country. One may infer from this that survival rates vary in different geographic regions of the U.S. although no study has compared survival for esophageal cancers across the continental U.S. Geographical analyses of other cancers have revealed differences amongst regions. Krupski et al. note that nonclinical factors associated with geography (e.g. income and ethnicity) play a role in determining whether men with early-stage prostate cancer undergo treatment with radiation or surgery or merely engage in watchful waiting [12]. In the analysis of ovarian cancer treatment, Polsky et al. found that the density of oncology hospitals, subject to geographic variation, predicts the use of chemotherapy [13]. Fairfield et al. also conducted an analysis of ovarian cancer treatment and found that geographic location impacts access to cancer-directed surgery, which is crucial to prognosis [14].

The aim of this study was to compare differences in the OS of esophageal cancer patients based on their geographic location in the continental USA, using Surveillance, Epidemiology, and End-Results (SEER). SEER collects individual patient data on the incidence, survival, and treatment of cancer [15]. As SEER collects data on factors putatively prognostic for survival, it is well suited to propose potential causes for the differences in survival rates in different geographic regions. While previous studies have examined the incidence of esophageal cancer in the U.S. as a whole, considering the influence of factors such as gender, age, race, and histology, none have studied the variation of survival in different geographic regions of the U.S. There exist health disparities that need to be addressed in different parts of the U.S., subject to socioeconomic, cultural, and environmental factors. To be able to achieve equity in cancer care across the U.S., these disparities in survival must be first identified. Here, we identify the geographic disparities in survival longitudinally, propose possible risk factors that may explain disparities and suggest measures that may address them. Hence, we sought to analyze differences in esophageal cancer survival by geographic region of the U.S. from the 1970s to the 2000s.

## Materials and methods

Raw data for this retrospective statistical analysis were obtained from the Surveillance Epidemiology and End Results Database (SEER), which is composed of a number of population-based registries, which together cover about 30% of the U.S. population. These registries regularly collect data on patient demographics, primary tumor site, tumor morphology, and stage diagnosis, first course of treatment, and follow-up for vital status [15]. Data Quality Profiles (DQP) are generated regularly for each registry, to ensure quality control [16]. The National Cancer Institute (NCI) does not require institutional review board approval for the use of SEER data. The limitations of SEER include a lack of standardization of medical records, comorbidities, treatment with chemotherapy, and information on risk factors [16].

**Figure 1:**
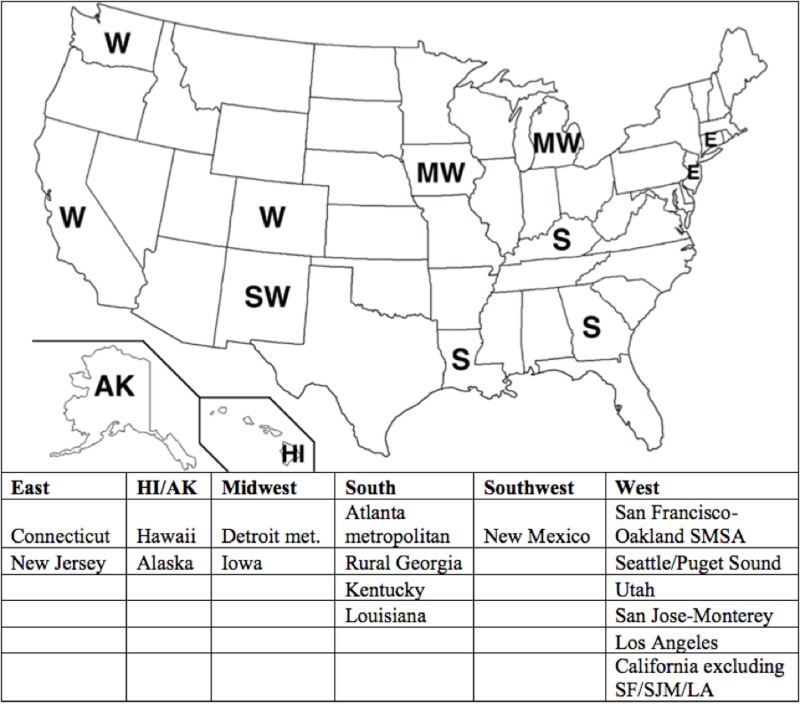
SEER Region Components of Geographic Regions E, east; HI/AK, Hawaii/Alaska; LA, Los Angeles; MW, midwest; S, south; SF, San Francisco; SJM, San Jose metropolitan area; SMSA, Standard Metropolitan Statistical Area; SW, southwest; W, west; SEER, Surveillance Epidemiology and End Results Database

The case listing session of the SEER*Stat 8.2.1 software package was used to identify patients diagnosed with cancer of the esophagus and gastric cardia via “primary site” codes C15.0-C16.0 from 1973-2009 (SEER data entry starts in 1973). Patients were stratified by decade of diagnosis (1973-1979, 1980-1989, 1990-1999, 2000-2009) and geographic region (East, Hawaii/Alaska (HI/AK), Midwest, South, Southwest, West). The map in Figure 1 shows the compositions and locations of the geographical regions within the U.S. [17]. To further characterize the differences between geographic regions, data were obtained on the number of NCI-designated cancer centers servicing each geographic region and the number servicing their constituent SEER registries [18]. Only centers that deliver care were included; basic laboratory centers were excluded. A cancer center was designated as serving a given registry if it was within a 160-kilometer radius of the territory encompassed by that registry, as indicated by Google Maps [19].To more accurately quantify the access to care amongst geographic regions, the number of centers per registry was averaged for each region. Calculations were also done to determine the patient burden of each center in a given region, by dividing the number of centers by the number of people within that region. The number of centers per square kilometer was similarly calculated by using U.S. census data on the size of the counties that comprised each of the SEER regions [20].

Other variables for analysis include patient age, gender, race, tumor stage, site, histology, therapy (radiation, surgery, or sequence of both), and metropolitan size. The stage at presentation was classified using the ‘localized,’ ‘regional,’ ‘distant,’ and ‘unstaged’ codes for “SEER historic stage A” (1973-1997) and “Summary Stage 2000 (1998+)” (1998-2009). Tumor site was grouped into distal (primary site codes ‘C15.2: abdominal esophagus,’ ‘C15.5: Lower thoracic portion,’ and ‘C16.0: Cardia, NOS.’) and non-distal. Histology was grouped into squamous, adenocarcinoma, or other based on “ICD-0-3 Hist/behav” codes.

For therapy, a value of ‘Surgery performed’ in “Reason no-cancer directed surgery” indicated surgery was performed, with other values indicating it was not. Codes for “Radiation” were used to determine if radiation was given, not given, or unknown. The values ‘Recommended, Unknown if administered,’ and ‘Unknown,’ meant it was unknown if radiation was given, the values ‘none’ and ‘refused’ indicated that radiation was not given while all other values indicated radiation was given.

The sequence of radiation with surgery (R&S sequence) was stratified into the following categories: 1. Post-operative RT, 2. Pre-operative RT, 3. Pre and Postoperative RT, 4. Intraoperative therapy (IORT), and 5. IORT combined with Pre or Post-op RT. Metropolitan size was split in to three categories using codes for “Urban-Rural continuum”: 1. counties in urban areas with a population > 1 million, 2: counties in metropolitan areas with a population between 250,000 and 1 million, and 3: counties in metropolitan areas with a population of < 250,000 and non-metropolitan counties.

The code “survival months” was used to determine the number of months a patient survived after diagnosis.

Statistical analysis

All data were analyzed using the SPSS version 22 (IBM, Armonk, NY, US) statistical software package. Results were considered statistically significant when P<0.05. Kaplan-Meier curves were used to estimate overall survival (OS), the endpoint for analysis, including five-year OS (% of a population with OS>60 months) and median OS (median number of months individuals in a given population survived). Univariate and multivariate analyses were conducted to identify differences in OS between the geographical regions of the U.S. and characterize other variables responsible for those differences. The univariate analysis used the log-rank analysis to compare OS for different values of a variable (e.g. median OS for Southern vs Eastern U.S). The multivariate analysis used an adjusted Cox Proportional Hazard regression analysis, including only those variables deemed statistically significant by the univariate analysis (P<0.05) to calculate the hazard ratio (HR). 95% Confidence Intervals (CI) were also calculated to evaluate the HR precision.

## Results

Study population and tumor characteristics

A total of 87,834 patients with cancer of the esophagus and gastric cardia were identified based on our inclusion criteria. The majority of the study cohort were males (N=66,492, 75.7%), who had a younger age of diagnosis (median age 65-69 years) relative to females (median age 70-74 years). Caucasians (N=72391, 82.5%) were diagnosed at an older age (median age 65-69 years) compared to African Americans (N=10552, 12.0 %, median age of diagnosis 60-64 years). The majority of the study cohort resided in urban areas with population > 1 million (N=53,906, 61.4%) while the remainder was split between counties in the metropolitan areas with populations between 250,000 and one million (N=16770, 19.9%), and counties with less 250,000 people (N=17021, 19.4%).

Table 1 shows the descriptive data of tumor characteristics and the treatment method utilization by geographic region. Roughly equal proportions of the population had a ‘local’ (24.4%), ‘regional’ (29.0%), or ‘distant’ (31.0%) stage disease. Most tumors were distally located (66.2%). The fraction of patients receiving radiation therapy (49.4%) was roughly equal to those who did not (48.5%). The majority of patients did not receive surgery (86.0%). Markedly more tumors were adenocarcinomas (53.9%) than those with a squamous histology (33.0%).

**Table 1:** Descriptive Characteristics of Tumors and Treatment Utilization by Geographic Region E, east; HI/AK, Hawaii/Alaska; MW, midwest; S, south; SW, southwest; W, west

		E	HI/AK	MW	S	SW	W	Total
Stage	Local	3811	534	4644	3313	755	8339	21396
	Regional	4568	691	4730	3767	656	11059	25471
	Distant	4633	789	5168	4007	627	11834	27058
	Unstaged	2540	232	2316	1848	318	6655	13909
Tumor Site	Distal	10117	1296	10920	8164	1586	26093	58176
	Non-distal	5435	950	5938	4771	770	11794	29658
Radiation	Yes	7628	1103	8978	6628	1018	18027	43382
	No	7745	1112	7407	5756	1321	19241	42582
	Unknown	179	31	473	551	17	619	1870
Surgery	Yes	2160	249	2659	1891	243	5056	12258
	No	13392	1997	14199	11044	2113	32831	75576
Histology	Adenocarcinoma	8457	938	8573	6549	1295	21513	47325
	Other	1957	203	2111	1619	397	5277	11564
	Squamous Cell Carcinoma	5138	1105	6174	4767	664	11097	28945
Total		15552	2246	16858	12935	2356	37887	87834

Survival outcomes by geographic region

**Figure 2:**
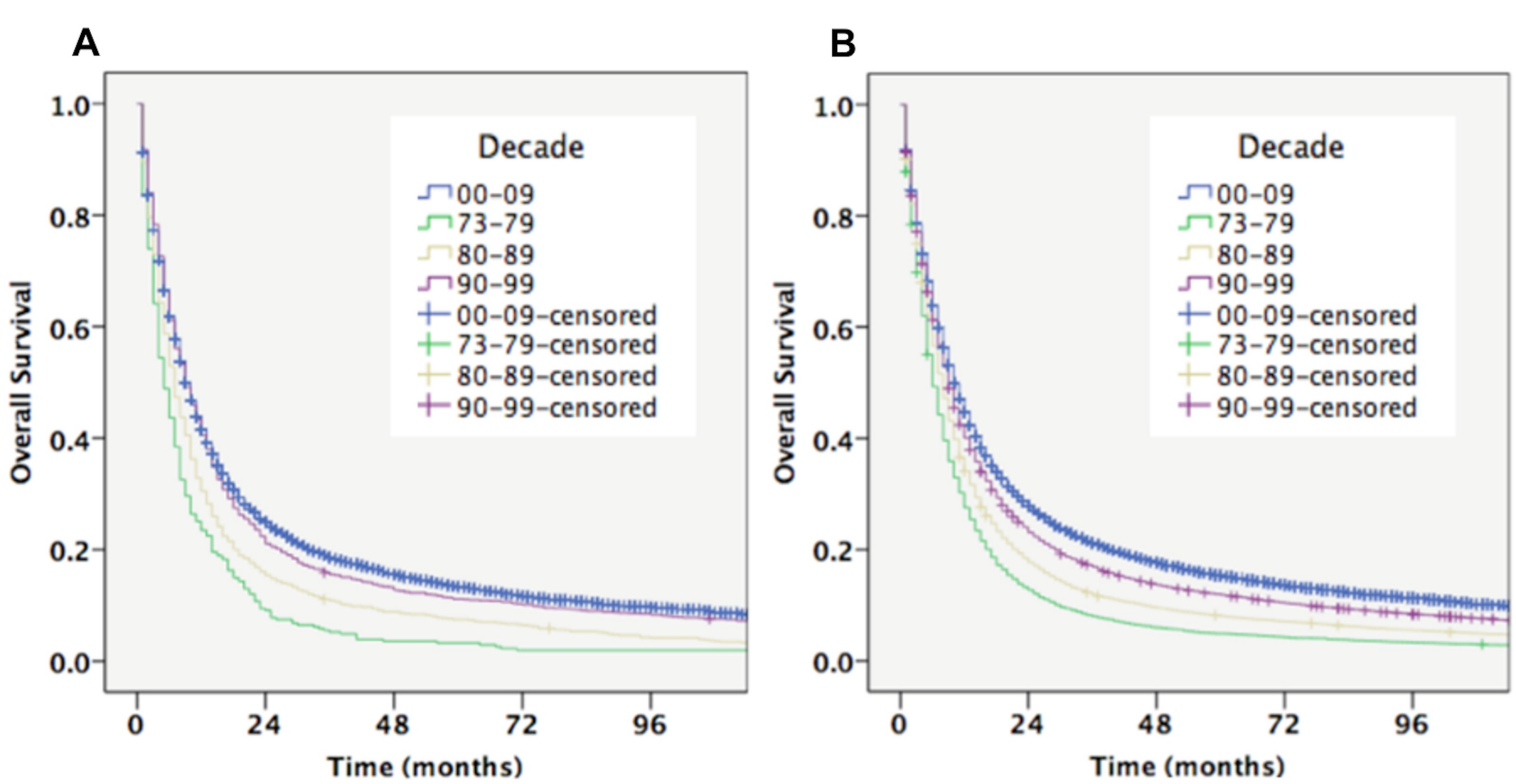
Overall Survival Comparison Between Southern and Eastern U.S. by Decade A. Overall survival in Southern U.S. by decade; B. Overall survival in Eastern U.S. by decade

The five-year OS improved from 4.9% (1970s) to 15.3% (2000s) of all cases and the median OS improved from 6.0 to 10.0 months (Table 2). Residence in the Southern U.S. was associated with a lower OS compared to other regions, from the 1970s (five months) to the 2000s (nine months) (P<0.001). Residence in the Eastern U.S. was prognostic of a better OS from the 1970s (six months) to the 2000s (12 months) (P<0.001). The difference in overall survival is compared in Figure 2.

**Table 2:** Overall Survival by Temporal Interval and Geographic Region OS, overall survival; E, east; HI/AK, Hawaii/Alaska; MW, midwest; S, south; SW, southwest; W, west.

		E	HI/AK	MW	S	SW	W	Median OS	5 year OS (%)	P-value
Decade	1973-1979	6	6	6	5	6	7	6	4.9%	<0.001
	1980-1989	8	8	8	7	7	8	8	8.1%	<0.001
	1990-1999	10	8	9	9	9	9	9	11.9%	<0.001
	2000-2009	12	10	11	9	10	10	10	15.3%	<0.001

Other impacts on OS

Considering all factors with P<0.001, the multivariate analysis (Table 3) revealed the following variables to be independent predictors of worse OS: increased age, African American race (Hazard Ratio = 1.160, P<0.001), distant disease (HR = 3.091, P<0.001), non-distal location (HR = 1.140, P<0.001), squamous histology (HR = 1.097, P<0.001), treatment without radiation (HR = 1.060, P<0.001), treatment without surgery (HR = 1.388, P<0.001), and residence in the Southern U.S. (HR = 1.150, P<0.001). The sequence of radiation and surgery was not prognostic. Residence in the Eastern U.S. was prognostic of a better survival compared to all the other regions (P<0.001).

**Table 3:** Factors Influencing Overall Survival: Multivariate Analysis CI, confidential interval; HI/AK, Hawaii/Alaska; IORT, intra-operative radiation therapy; RT, radiation therapy; R&S Sequence, sequence of radiation with surgery

		P-value	Hazard Ratio	95.0% CI	
				Lower	Upper
Age	<19 years (reference)				
	20-24 years	0.008	2.317	1.241	4.325
	25-29 years	0.069	1.713	0.960	3.057
	30-34 years	0.025	1.887	1.082	3.294
	35-39 years	0.038	1.790	1.033	3.102
	40-44 years	0.008	2.092	1.211	3.612
	45-49 years	0.003	2.268	1.315	3.912
	50-54 years	0.003	2.267	1.315	3.908
	55-59 years	0.002	2.332	1.353	4.020
	60-64 years	0.001	2.493	1.447	4.297
	65-69 years	0.001	2.623	1.522	4.521
	70-74 years	<0.001	2.909	1.688	5.013
	75-79 years	<0.001	3.290	1.909	5.671
	80-84 years	<0.001	3.898	2.261	6.719
	>85 years	<0.001	4.838	2.806	8.341
Race	African American (reference)				
	Caucasian	<0.001	0.739	0.620	0.881
	Other	<0.001	1.160	1.133	1.187
	Unknown	<0.001	0.866	0.834	0.899
Decade	2000-2009 (reference)				
	1973-1979	<0.001	1.590	1.546	1.636
	1980-1989	<0.001	1.314	1.286	1.343
	1990-1999	<0.001	1.180	1.159	1.202
Region	East (reference)				
	HI/AK	<0.001	1.144	1.083	1.207
	Midwest	<0.001	1.058	1.033	1.084
	South	<0.001	1.150	1.120	1.180
	Southwest	<0.001	1.182	1.128	1.238
	West	<0.001	1.051	1.030	1.073
Tumor site	Distal (reference)				
	Non-distal	<0.001	1.140	1.119	1.161
Histology	Adenocarcinoma (reference)				
	Squamous Cell Carcinoma	<0.001	1.097	1.074	1.119
	Other	<0.001	1.054	1.031	1.079
Stage	Local (reference)				
	Regional	<0.001	1.569	1.537	1.602
	Distant	<0.001	3.091	3.027	3.156
	Unknown	<0.001	1.658	1.618	1.698
Surgery	Yes (reference)				
	No	<0.001	1.388	1.203	1.601
Radiation	Yes (reference)				
	No	<0.001	1.060	1.043	1.078
	Unknown	<0.001	1.141	1.086	1.199
R&S Sequence	IORT & Pre or Post-op RT (reference)				
	Pre-operative RT	0.083	0.879	0.760	1.017
	Pre & Post-op RT	0.033	1.272	1.020	1.586
	IORT	0.244	0.716	0.408	1.256
	Post-operative RT	0.973	0.998	0.863	1.153

As shown in Table 4, the West and the East had the highest amount of cancer centers (12 and seven, respectively) and centers per registry (3.2 and 3.5, respectively). The Southern U.S. and HI/AK had the least amount per registry (0.75 and 0.5, respectively). The South also had the lowest number of centers per person (1.6E-07) while the East had the most (5.7E-07).

**Table 4:** Number of NCI-designated Cancer Centers by Geographic Region HI/AK, Hawaii/Alaska; NCI: National Cancer Institute

Region	East	HI/AK	Midwest	South	Southwest	West	P-value
Total	7	1	3	2	1	12	<0.001
Per registry	3.5	0.5	1.5	0.75	1.0	3.2	<0.001
Per person	5.7E-07	6.8E-07	4.3E-07	1.6E-07	4.9E-07	2.7E-07	<0.001
Per sq km	2.2E-04	6.7E-07	2.0E-05	8.8E-06	3.2E-06	1.7E-05	<0.001

## Discussion

As our data indicate, esophageal cancer survival has improved from the 1970s to the 2000s in all parts of the U.S., both in terms of five-year OS (4.9% to 15.3% of patients) and the median OS (6.0 to 10.0 months). Our multivariate analysis showed that residence in the Eastern U.S. was prognostic for a better survival outcome relative to all the other regions (P<0.001). The impact of the geographic region is not as significant as known prognostic factors, such as disease stage and treatment (Table 3). Survival was better for those treated with surgery (HR = 1.388, P<0.001) compared to those not treated, which may be partially explained by a better prognosis for those with the surgically resectable disease.

This variation in survival based on geography may be either due to the unbalanced distribution of medical resources or the regional difference of cancer biology. Our analysis found that the non-distal tumor location and squamous histology were an independent predictor of worse overall survival (Table 3). It is worth noting that in our study cohort, people in the West and the East had a relatively high percentage of distally located tumor (69% and 65%, respectively, P<0.001) and a low percentage of squamous carcinoma (29% and 33%, respectively, P<0.001), as opposed to other regions, such as HI/AK and South (Table 1), which may, at least partially, contribute to the relatively higher OS in these two areas (Tables 2-3) and may suggest a need to devote specific treatment and research resources proportionally by regional tumor biology in the future. In the meantime, consistent with other literature [21], the worse OS in African Americans (H = 1.160, P<0.001) (Table 3) may be attributed to a high proportion of squamous histology (74%, P<0.001) (data not shown) as well.

The rate at which improvements in care are implemented may vary by geographic region and may be a function of socio-economic disparities and/or of the predominance of race and ethnicity. Since our analysis is indicative of better survival in the Eastern and Western U.S. regions (as compared to the Southern regions) (Tables 2-3), one may conclude that the implementation of improvements in the management of esophageal cancer may be relatively slow in the South, serving as an impetus for us to delve further.

Greater access to advanced, and high-quality care, as indicated by the presence of more NCI-designated cancer centers in the Eastern (N=7) and Western (N=12) U.S. (Table 4) may be one of the determining factors for their comparatively better survival. In comparison, the Southern U.S. with only two NCI-designated cancer centers overall, the lowest number of centers per person (1.6E-07, resulting in a high patient burden) and only 0.75 cancer centers per registry (lower than any region other than HI/AK) (Table 4), had poorer survival relative to the East, West, and Midwest. Only a single center serves the Atlanta and Rural Georgia registries while none serve Louisiana. Allocation of more resources to the Southern U.S. and providing optimal accessibility to advanced care may help improve such dismal results.

In addition to our characterization of the factors reported by SEER, an analysis of geography-associated factors reported in the literature may provide further insight into disparities in survival. Many studies have noted that treatment outcomes could be influenced by travel time and that access to chemotherapy was subject to the availability of local and visiting oncologists [22-24]. In our study, the poor survival in the largely rural Southern U.S. may be explained by this lack of access to basic care in rural areas, in addition to a dearth of advanced NCI centers (Table 4). Local-scale intervention may be the most efficient, immediate means to increase the rate of improvement in survival. The use of Visiting Consulting Clinics (VCCs) is well-suited for this purpose, having increased access to oncologists in rural areas [23-24]. A follow-up to our current study may investigate VCC usage in the Southern U.S. to recommend potential areas of implementation for improving access to care. Geographic location is also a determinant of socioeconomic status, which plays a significant role in access and quality of care, and is associated with risk factors for esophageal cancer such as obesity, tobacco, and alcohol usage [25-27]. Obesity, correlated with a two-fold worsening of disease-specific, disease-free and overall survival in EAC patients, is a significant emerging risk factor [28]. Shapiro et al. noted that cigar smoking resulted in an increased risk of death for esophageal cancer patients [29]. Lastly, high levels of alcohol consumption were attributed to a major cause of exceptionally high esophageal cancer mortality amongst African Americans in Washington DC [30].

Our study is limited in that it was retrospective and could not account for comorbidities or the utilization of chemotherapy, as SEER does not record this data. Comorbidities play a significant role in mortality and, hence, could have confounded some of our results. Our findings on disease stage may also have some errors because of technological improvements, including PET/CT that have increased the precision of staging, thereby producing the phenomenon of stage migration. Stage migration refers to the reclassification of patients into different prognostic groups (e.g. from localized to regional disease), thereby changing survival rates in each prognostic group, without changing individual patient outcomes. For instance, what may have appeared to be localized disease in the 1970s may actually be regional. Another limitation of SEER is that it only categorizes race as Black, White, other, or unknown. Globally, esophageal cancer is most common in Eastern Asian countries, but we were unable to see if rates of esophageal cancer in Americans of Eastern Asian race are also high, as they are classified as “other.” This suggests that a future study on esophageal cancer incidence with data disaggregating Asian and Pacific Islander data into constituent subgroups (e.g. Eastern Asian) may merit consideration. However, the conclusions drawn regarding patients in the continental U.S. are likely valid, as the trends described are largely consistent through the entire four decades of the study.

## Conclusions

There exist disparities in esophageal cancer survival and quality of care through different geographic regions of the U.S., which may be attributed to a combination of the unbalanced distribution of medical resources, regional difference of cancer biology, and other lifestyle and socioeconomic factors, and should merit the consideration of shifting resources to regions, as necessary, to address the unique circumstances of each. The variables we analyzed provide a partial explanation of the differences among geographic regions. Further research can be done to identify other variables responsible, investigate factors that interplay with geography, such as socioeconomic status, and determine the means of implementing improvements in access to and quality of care.
